# Increased Blood Flow Velocity in Middle Cerebral Artery and Headache Upon Ingestion of Ice Water

**DOI:** 10.3389/fneur.2019.00677

**Published:** 2019-06-28

**Authors:** Ole Hensel, Philipp Burow, Stephan Mages, Andreas Wienke, Torsten Kraya, Stephan Zierz

**Affiliations:** ^1^Department of Neurology, Martin Luther University, Halle, Germany; ^2^Institute of Medical Epidemiology, Biostatistics, and Informatics, Martin Luther University, Halle, Germany

**Keywords:** ice cream headache, ingestion of cold stimuli, mean flow velocity, cerebrovascular resistance, trigeminal-parasympathetic vasodilator reflex

## Abstract

**Introduction:** “Headache attributed to ingestion or inhalation of a cold stimulus” (HICS) is one of the most common primary headache disorders. Little is known about the pathophysiology of HICS and other headache disorders. The aim of this study was to analyze mean flow velocity (MFV) and cerebrovascular resistance (RI) in both middle cerebral arteries (MCA) upon ingestion of ice water.

**Methods:** The MFV and RI in both MCAs was continuously measured by transcranial sonography. HICS was induced by drinking 200 ml of ice water.

**Results:** In all volunteers, the ingestion of ice water led to a decrease in RI, which was accompanied by an increase in MFV. In volunteers with induced HICS, MFV were significantly higher compared to volunteers that did not experience HICS. In volunteers with HICS, MFV increased even more significantly when lacrimation occurred compared to volunteers in which it did not. In volunteers without induced HICS, MFV was higher in those volunteers with a positive history of HICS than in those with a negative HICS history.

**Conclusion:** This study revealed a raised MFV upon ingestion of ice water. Volunteers with a provoked case of HICS had a higher MFV than volunteers without HICS. The increase in MFV was even higher when the headache was accompanied by lacrimation. This may indicate an involvement of the trigeminal-parasympathetic vasodilator reflex.

## Introduction

Headache after ingestion of a cold stimulus (HICS) is colloquially called an ice cream headache. The recent International Headache Classification (ICHD-3, 4.5.2) describes this as a “short-lasting frontal or temporal pain, which may be intense, induced in susceptible people by passage of cold material (solid, liquid, or gaseous) over the palate and/or posterior pharyngeal wall” (1). The prevalence of HICS in students is 62% and decreases in adults to 22–36% (3). The experimental stimulation of the trigeminal nerve due to the ingestion of an ice cube, provoked a headache in 17% of healthy adults, in 23–32% of patients with tension-type headache and in 48–74% of patients with migraine (4, 5). In 13 of the volunteers, drinking ice water induced a non-significant increase in mean flow velocities (MFV) in the anterior cerebral artery but not in the middle cerebral artery (MCA) (2). In two out of three volunteers, headache upon ingestion of ice cream lowered the MFV in the MCA (6). The data on changes in MFV in the MCA induced by the ingestion of cold stimuli are inconsistent.

At present, the pathogenesis of HICS is unknown. The aim of this study was to analyze the blood flow velocity and cerebrovascular resistance in both MCA upon ingestion of ice water.

## Materials and Methods

### Patients

The study examined 77 healthy volunteers who were recruited through advertisement on a university billboard and social media. Ages ranged between 18 and 60 years (mean age 26.5 ± 7.7 years; 47/77 women). According to ICHD-3, all volunteers included in the study had no additional primary headache disorder. Exclusion criteria included pregnancy, cerebrovascular disease, brain tumor, or history of syncope.

### Study Protocol

**Figure 1:**
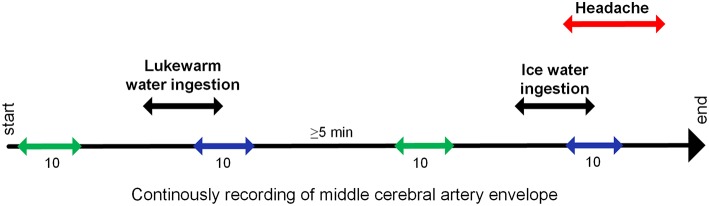
Study protocol: The velocity envelope of both middle cerebral arteries was continuously recorded. Start and end of ingestion and headache was marked in the recording. For analyses, this velocity data were averaged for resting period 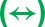 and end of stimulation period 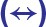. The durations of these periods are given in heartbeats.

In order to assess confounding factors, the volunteers were told to drink 200 ml of luke-warm water (temperature 22 ± 2°C) as fast as possible. After 5 min, they were told to drink 200 ml of ice water (temperature 0°C) as fast as possible (Figure 1). The volunteers indicated HICS by raising their thumb. Volunteers were then asked about study-related symptoms (e.g., lacrimation, rhinorrhea, and headache intensity) by way of a questionnaire.

### Blood Flow Measurements

Using a self-constructed holding device (“Hallesche Halterung”), bilateral transcranial Doppler probes (2 MHz, Multidop X4®, DWL, Sipplingen, Germany) were fixed onto a temporal bone with a sufficient acoustic window. The blood flow envelope of the bilateral MCA was simultaneously and continuously recorded at a depth of 50 mm. The start and end of water ingestion and provoked HICS were marked in the recording.

### Analysis of Data

The MFV envelope, with data points every 10 ms, were exported along with the marks as a text file and then imported into the OriginPro® software 9.1.0G (OriginLab Corporation, Northampton, USA); velocity outliners were manually removed. A self-programmed procedure detected the end-diastolic velocity and the peak-systolic velocity in the envelope, and calculated the MFV and RI in both MCAs. The RI was used as a parameter for cerebrovascular resistance, which is mainly controlled by resistance vessels. The MFV and RI in the MCA were averaged for resting and end of stimulation. Resting period was defined as 10 heartbeats and ended 10 heartbeats before the start of stimulation. The end of the stimulation period was defined as the last five heartbeats before and after drinking stopped (Figure 1). The heart rate (HR) and RMSSD (root mean square of successive differences: parasympathetic influence on heart rate variability) was calculated from the time between two systolic peaks and averaged for resting and end of stimulation period. The procedure automatically calculated the changes in relative percentages at the end of stimulation compared to the resting period.

All data are reported as mean ± SD or 95% confidence intervall. Paired or two-sample *t*-tests were used and alpha = 0.05 was taken as the significant level. The study was approved by the ethics committee of the Faculty of Medicine at Martin Luther University Halle-Wittenberg and followed the ethical standards of the Helsinki Declaration of 1975, as revised in 2013. All participants provided informed and written consent before taking part.

## Results

### Ingestion of Luke-Warm Water

No HICS or eye tearing was provoked by this stimulus. There was no difference between resting period and luke-warm water ingestion in terms of MFV and RI (2.8 ± 8.8%; −5.1 ± 8.0%; both *p* > 0.05). However, HR increased upon ingestion of the luke-warm water (18.3 ± 14.4%, *p* < 0.001).

### Ingestion of Ice Water

Ice water provoked a mostly stabbing bilateral HICS in 39/77 (48%) volunteers (median latency 20 ± 19 s, duration 12 ± 10 s, pain intensity was 4.5 ± 2.2 of 10 on a numerical pain scale). In 10 of 39 (26%) after the first HICS (HICS resolved completely) a second headache occurred with a latency of 12 s (range 3–26 s). This and other clinical details of the experiment have been published elsewhere (7). At the end of ice water ingestion, the MFV in both MCAs had increased compared to luke-warm water stimulus (7.0 ± 10.2%, *p* < 0.001). The HR was higher than during the resting period (8.3 ± 20.2%, *p* < 0.001) but lower than with the luke-warm water stimulus (*p* < 0.005). The increase in RMSSD was higher compared to luke-warm water stimulus (169.0 ± 330.2 vs. 27.0 ± 127.7%; *p* < 0.001).

**Figure 2:**
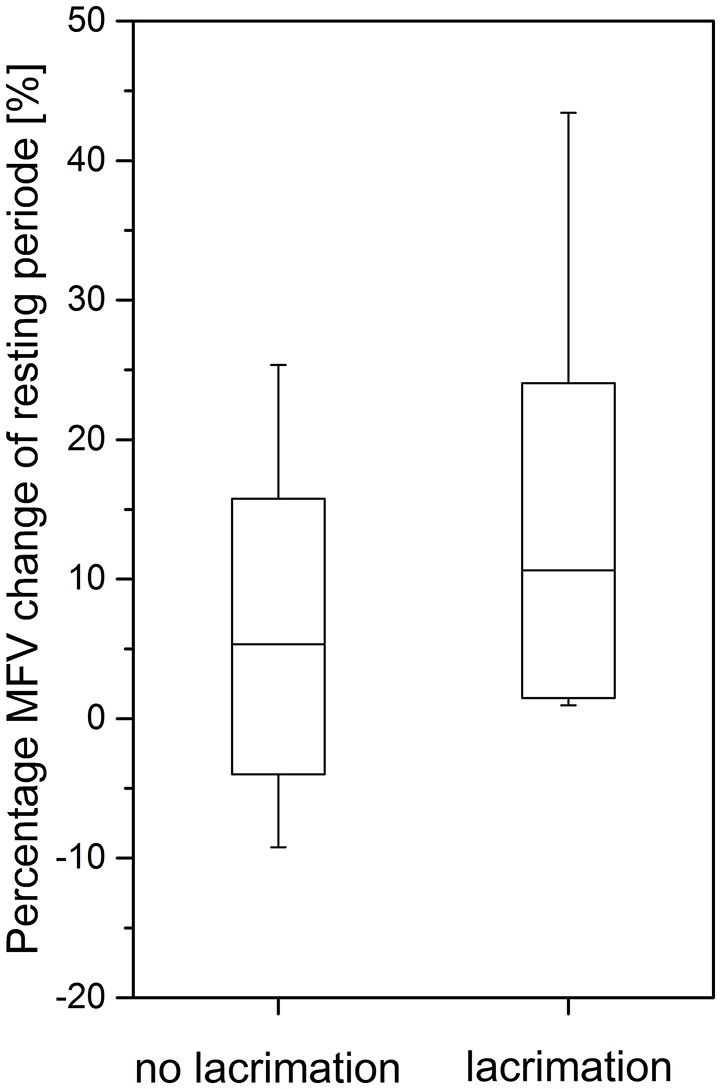
The ice water induced change of mean flow velocity (MFV) in middle cerebral artery (MCA, median ± standard deviation, 95% Confidence interval) in volunteers with and without lacrimation upon HICS (significant difference *p* = 0.006).

Volunteers with HICS had higher MFV rates than volunteers without HICS (9.3 ± 11.1 vs. 4.6 ± 8.6%, *p* = 0.003, Table 1). There was no difference in HR (11.0 ± 17.9 vs. 5.7 ± 22.0%, *p* = 0.250). Lacrimation after ice water ingestion occurred in 26/77 (34%) volunteers. Half of the volunteers with ice water induced HICS (19/39 volunteers, 49%) experienced lacrimation. In volunteers with HICS and lacrimation, the increase in MFV was even more pronounced than in volunteers with HICS but without lacrimation (12.8 ± 11.3 vs. 5.9 ± 9.9%, *p* = 0.006; Figure 2). The pain intensity (range 0–10 on a numerical pain scale) did not differ between volunteers with and without lacrimation (4.1 ± 2.7 vs. 4.6 ± 2.2%, *p* = 0.512).

**Table 1:** Cerebrovascular changes due to luke-warm and ice water ingestion (*n* = 77); mean in % (with 95% confidence interval).

	**MFV**	**RI**	**HR**	**RMSSD**
**Luke-warm water stimuli**	2.8 (1.4 to 4.2)	−5.1 (−6.4 to −3.8)	21.3 (18.0 to 24.6)	27.0 (−2.0 to 56.0)
**Ice water stimuli**				
No provoked HICS (*n* = 38)	4.6 (2.6 to 6.5) *p* = 0.184^*^	−7.3 (−9.1 to −5.5) ***p*** **<** 0.042^*^	13.1 (7.3 to 19.0) ***p*** **<** 0.005^*^	116.6 (54.2 to 178.9) ***p*** **<** 0.005^*^
Without HICS history (*n* = 28)	3.9 (1.7 to 6.1)	−6.6 (−8.4 to −4.8)	14.0 (8.4 to 19.6)	107.3 (32.9 to 181.7)
With HICS history (*n* = 10)	6.6 (2.2 to 11.1)	−9.4 (−14.4 to −4.3)	10.8 (−7.6 to 29.2)	142.4 (5.4 to 279)
With provoked HICS (*n* = 39)	9.3 (6.8 to 11.7) ***p*** **<** 0.001^*^ p **<** **0.004#**	−6.6 (−8.4 to −4.7) *p* < 0.234^*^ p < 0.597#	8.5 (0.9 to 16.2) ***p*** **<** 0.002^*^ p < 0.336#	220.0 (83.4 to 356.6) ***p*** **<** 0.011^*^ p < 0.171#

HICS, Headache attributed to ingestion or inhalation of a cold stimulus; HR, heart rate; MFV, mean flow velocity in both middle cerebral arteries; RI in both middle cerebral arteries; RMSSD, root mean square of successive differences;

* different to luke-warm water stimuli by paired t-test;

# *different to volunteers without HICS by two sample t-test; bold values indicate significant difference*.

In volunteers without provoked HICS, MFV was higher in volunteers with a positive HICS history than in volunteers with a negative HICS history (Table 1).

## Discussion

The ingestion of ice water leads to a lowering of cerebrovascular resistance and an increase in MFV in the proximal MCA. The increase in MFV was most pronounced in volunteers with provoked HICS.

Ingestion of ice water stimulates cold receptors of the trigeminal (V2, V3), glossopharyngeal and vagus nerves. The trigeminal nerves might activate the trigeminocervical complex and subsequently the inferior and superior salivatory nuclei. The resulting parasympathetic projection to the lacrimal gland and cranial arteries might cause tearing and reduction in cerebrovascular resistance. The resulting dilation of downstream resistance vessels might cause the MFV to increase. The increased heart rate variability (RMSSD) is due to an increase in parasympathetic activity. The cold stimulation of the trigeminal nerve might also activate the sympathetic system, which causes peripheral vasoconstriction, a lowering of skin temperature (8) and increase in MAP (9).

The diminished cerebrovascular resistance may be caused by antidromic activation of the trigeminal fibers and neurogenic inflammation.

It has been identified that drinking luke-warm water simultaneously increases parasympathetic and sympathetic activity in humans (10). The two activities may interact upon ingestion of ice water and provoke the cardiovascular effects. This interplay may explain why ingesting ice water increases the HR compared to the resting period but why heart rates are lower than when ingesting luke-warm water.

Volunteers that did not experience HICS also showed a significant MFV increase; therefore, the increase in MFV in healthy volunteers due to the ingestion of cold stimuli appears to be a physiological phenomenon. The cardiovascular effects that were provoked and observed were similar to the diving reflex. The diving reflex conserves oxygen by redirecting blood flow from the extremities and skin to the heart and brain. Such a redirection of blood to the brain might be useful in clinical situations, such as ischemic stroke (11).

The increase in MFV was more pronounced in volunteers with provoked HICS than in volunteers without HICS. However, volunteers with a positive HICS history but negative HICS provocation had a moderate MFV increase. This indicates that a positive HICS history is associated with an increased sensitivity to cold ingestion and subclinical increase in MFV even without HICS actually being provoked.

An association with HICS, lacrimation, and the pronounced increase in MFV and decrease in RI indicates that vascular changes are important in the pathogeneses of this phenomenon. However, it remains unclear which mechanisms (parasympathetic or trigeminal system) and which vasoactive molecules (e.g., VIP, CGRP, and substance P) are responsible for the clinical and vascular observations.

## Data Availability

The datasets generated for this study are available on request to the corresponding author.

## Ethics Statement

This study was carried out in accordance with the recommendations of ethics committee of the Faculty of Medicine at Martin Luther University Halle-Wittenberg with written informed consent from all subjects. All subjects gave written informed consent in accordance with the Declaration of Helsinki. The protocol was approved by the ethics committee of the Faculty of Medicine at Martin Luther University Halle-Wittenberg.

## Author Contributions

OH: substantial conception and design of the paper, the acquisition, analysis, and interpretation of data for the paper. PB: critical revision of the paper with respect to important intellectual content. SM: conception and design of the paper and data acquisition for the paper. AW: substantial contributions to the design of the paper and analysis of data for the paper. TK: substantial conception and design of the paper, interpretation of data for the paper, and critical revision of the paper with respect to important intellectual content. SZ: substantial conception and design of the paper, interpretation of the data for the paper, and revising it critically for important intellectual content.

### Conflict of Interest Statement

The authors declare that the research was conducted in the absence of any commercial or financial relationships that could be construed as a potential conflict of interest.
